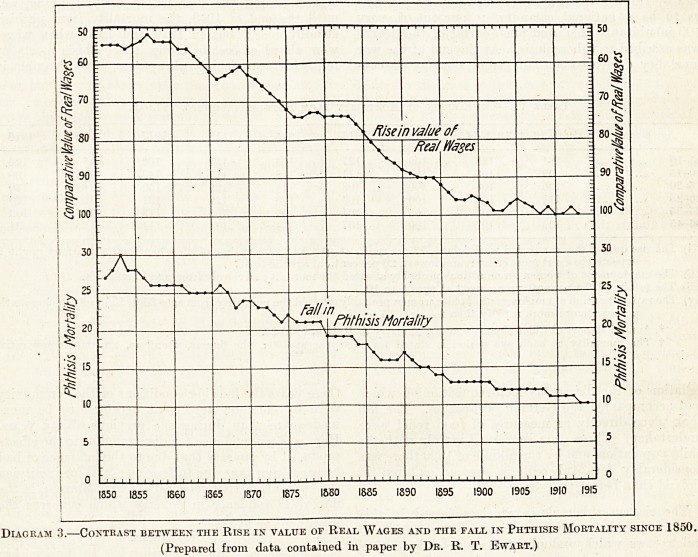# The Decline and Fall (?) of Tuberculosis.—II

**Published:** 1922-05

**Authors:** Edgar L. Collis

**Affiliations:** (Mansel Talbot Professor of Preventive Medicine, Welsh National School of Medicine)


### THE DECLINE AND FALL (?) OF TUBERCULOSIS.—II.

By EDGAR L. COLLIS, M.A., M.D., M.R.C.P. (Mansel Talbot Professor of Preventive Medicine, Welsh National School of Medicine.)

**Incidence of Tuberculosis.**

The matter may be carried further by examining the varying incidence of tuberculosis in towns and in country, and in the two sexes. Brownlee[6] has shown reason for considering that, at any rate in London, tuberculosis was not very prevalent in the first half of the eighteenth century, but that in the last half it became more prevalent, rising synchronously with the great industrial development which then saw its birth. This increase in an infectious and endemic disease is what might be expected when the population became more closely aggregated, and so more exposed to risk. The height of the wave was passed about 1820, and thereafter the disease has declined. This decline, on the immunity theory, might be due to the infection, which must by then have become ubiquitous, vaccinating the population against itself; but during the nineteenth century the growth and creation of industrial centres, with influx of persons from agricultural areas, which had commenced in the latter half of the eighteenth century, proceeded at an unprecedented rate, and was associated with vastly increased means of communication. Indeed, every influence for multiplying exposure to risk of infection was multiplied. Yet the disease decreased. If this decrease were due to immunisation, that part of the population should have benefited most which was most exposed to risk—*i.e.*, the urban districts, and that part of the population in those districts longest exposed to risk—*i.e.*, the older persons. These points can be investigated by examining the prevalence of the disease since 1850, that is for the period during which statistical records are reasonably reliable.

A steady and marked fall is found to have taken place (*see* Table II.), which has been more pronounced for females than for males. The fall has taken place in both sexes at all ages, but has been more pronounced in early adult life (*see* Tables IV. and V.).

Comparison of a closely populated industrial area with a distinctly rural area (*see* Diagrams 1 and 2) brings out that the fall has occured just as much in the one as in the other, at all ages and in both sexes. The least fall has taken place among the industrial males after middle life. So that we find the exact contrary has been taking place to what was postulated should take place if the decrease were due to immunisation. Hence, we are driven to consider that the decline in tuberculosis is not so much a matter of immunity acquired through the influence of an ubiquitous infection, as of an increase in the natural power of resisting the disease. And, incidentally, of resisting disease in general, since simultaneously the general death rate has been falling (*see* Table II.).

If this conclusion be accepted, consideration must be given to the question which calls for an answer—viz., to what influence or influences has the fall in tuberculosis which has occurred been due? Clearly the link in the chain of the causation of tuberculosis is weakening somewhere. If an answer can be given, then efforts to accentuate these influences, or the main influence, may be expected to bring the disease under control; for experience teaches that if a link in the chain of disease-causation can be sufficiently weakened it need not be absolutely broken for control to be obtained. Leprosy in Norway, attacked by incomplete segregation, is an instance in point; while mosquitoes, lice and fleas still exist in the land, even though in diminished numbers; but malaria, typhus and scarlet fever are practically under control.

TABLE V.—COMPARATIVE DEATH-RATE FROM PHTHISIS AT CERTAIN AGES.

Male and Female.*

| Period. | 10–15. | | 15–20. | | 45–55. | | 55–65. | |
|---|---|---|---|---|---|---|---|---|
| | M. | F. | M. | F. | M. | F. | M. | F. |
| **ENGLAND AND WALES.** | | | | | | | | |
| 1851–1860 | 100 | 169 | 100 | 147 | 100 | 82 | 100 | 72 |
| 1861–1870 | 80 | 137 | 92 | 130 | 101 | 75 | 99 | 62 |
| 1871–1880 | 63 | 111 | 70 | 100 | 101 | 64 | 96 | 53 |
| 1881–1890 | 45 | 92 | 54 | 75 | 91 | 54 | 87 | 45 |
| 1891–1900 | 31 | 66 | 41 | 54 | 82 | 43 | 78 | 37 |
| 1901–1910 | 22 | 52 | 32 | 41 | 72 | 34 | 71 | 31 |
| **LONDON.** | | | | | | | | |
| 1881–1890 | 38 | 70 | 49 | 50 | 143 | 67 | 139 | 54 |
| 1891–1900 | 26 | 54 | 40 | 40 | 133 | 59 | 130 | 47 |
| 1901–1910 | 20 | 43 | 33 | 33 | 114 | 47 | 115 | 42 |
| **LANCASHIRE.** | | | | | | | | |
| 1881–1890 | 55 | 94 | 64 | 78 | 106 | 61 | 97 | 52 |
| 1891–1900 | 38 | 69 | 45 | 55 | 101 | 48 | 87 | 39 |
| 1901–1910 | 28 | 58 | 35 | 43 | 95 | 41 | 92 | 36 |
| **BEDFORDSHIRE.** | | | | | | | | |
| 1881–1890 | 32 | 104 | 29 | 75 | 69 | 54 | 52 | 45 |
| 1891–1900 | 17 | 44 | 31 | 43 | 60 | 44 | 53 | 41 |
| 1901–1910 | 23 | 49 | 30 | 37 | 50 | 24 | 46 | 36 |
| **SUSSEX.** | | | | | | | | |
| 1881–1890 | 36 | 73 | 44 | 67 | 93 | 53 | 89 | 45 |
| 1891–1900 | 23 | 59 | 39 | 50 | 79 | 42 | 77 | 35 |
| 1901–1910 | 17 | 47 | 28 | 38 | 66 | 28 | 66 | 28 |

* This Table is abstracted from the Annual Report of the Chief Medical Officer of the Ministry of Health, 1919–20. Appendix V. The death rate for males at each age period for 1851–1860 is stated as 100; the columns, therefore, are only comparative when read from above downwards.

**What Influences have been at Work ?**

The period since 1850 has been one during which many social alterations have taken place, and a close correlation could be established between many of them and the decline in the phthisis mortality. Such things as the introduction of municipal hygiene, however, which was embarked upon from 1874 onwards, must be put on one side, since the disease started to decline at least thirty years earlier, and afterwards continued to decline at the same rate, neither faster nor slower. Karl Pearson[7] has wittily pointed out that correlations could be shown to exist between this falling mortality and an increase in the consumption of bananas or in the reading of newspapers; similarly, increase in railroads or in the building of ships could be used for the purpose. Each, of these things, however, has either been caused by or has been an element in the causation of, a steady and rapid increase in wealth. Now one thing upon which all observers are agreed is that tuberculosis, as an endemic disease, is a *pestis pauperum*. So much is this so that Newsholme[5] has written that " the

Diagram 1.—Phthisis Death Rate in Devonshire.

Diagram 2.—Phthisis Death Rate in Lancashire.

correspondence between the variations of phthisis and those of pauperism has been so marked as to justify the use of the figures of total pauperism as approximate indexes of the total amounts of phthisis when the actual phthisis figures cannot be had."

**Poverty.**

The matter may be examined by comparing the movements which have taken place since 1850 in the value of real wages and in phthisis mortality (*see* Diagram 3). The similarity is remarkable; even the steadying of the rise in real wages from 1900 onwards is associated with a steadying in the fall of phthisis. One influence of poverty in relation to disease which calls for special consideration is food supply. The investigations of Booth, Rowntree and others have shown that a large part of our population has existed on the verge of semi-starvation. Medical sociologists have for long pointed to the undue prevalence of the disease among this portion of the community, and have ascribed the prevalence to underfeeding. This point of view has been expressed by Kinloch[4]: "Even with our strictly limited knowledge of immunological processes, it is clear that there is for each disease what, for want of a better terminology, may be described as a 'proper' metabolism." Some explanation of how proper metabolism is maintained is forthcoming from recent work upon food accessory factors or vitamins, and their influence upon so-called deficiency diseases. The presence of these substances in food is necessary for absorption to take place; and the more energy is being expended the greater must be the supply of vitamins as well as of the energy-carrying constituents[16].

**The War Period.**

An uncontrolled but nevertheless a particularly valuable experiment was presented during the Great War. All over the world, but particularly in the belligerent countries, there arose a food shortage which became more and more stringent from year to year. Simultaneously there occurred a rise in tuberculosis mortality, which by 1918 amounted in the United States, where the food position was least affected, to 5·7 per cent., in Great Britain, where the food position was more stringent, to 27·6 per cent., and in Central Europe, where something approaching to national starvation prevailed, to 67 per cent.[8]. Professor Selter has no hesitation in tracing this rise to underfeeding; and we know that in Central Europe, while all foodstuffs were scarce, the scarcity of fats was most pronounced. This rise, however, in Germany hardly affected the first year of life; while

Diagram 3.—Contrast between the Rise in value of Real Wages and the fall in Phthisis Mortality since 1850.
(Prepared from data contained in paper by Dr. R. T. Ewart.)

in this country the infant tuberculosis rate absolutely fell. A possible explanation is that breast-feeding is more generally resorted to when food, and especially milk, is scarce. While this rise was taking place in the general community, the prevalence of tuberculosis was particularly low among the fighting forces, who were well fed, although they were otherwise exposed to hardship and severe mental strain. When food became more plentiful on the cessation of hostilities tuberculosis mortality immediately declined. Professor Möllers states for Germany that in the first half of 1914 it was 1·75 per 1,000, in the first half of 1919 it was 3·44, and in the first half of 1920 it was 2·08.

The position in Poland was even more marked. In that country before the war, Warsaw could boast of a lower tuberculosis rate than Paris, Vienna or Moscow. But by 1917 the disease had reached such proportions as to be a national calamity[9]; four out of every 100 inhabitants died, and one-quarter of the deaths was ascribed to tuberculosis. At the end of the war mortality decreased actually from the day of cancellation of the disastrous food-requisitioning which had accompanied the German occupation. The fall took place directly as measures of food relief were undertaken. These were concerned mainly with the child population, and by the middle of 1920 there was considerably less tuberculosis among children in Poland than before the war.

The rise in tuberculosis which occurred in this country has been ascribed to the deterioration in milch cows which undoubtedly set in early during the war; certainly the position in relation to the supply of milk and butter, as the war progressed, became increasingly stringent.

The rise among females during this period has been shown by Greenwood[10] after careful study to have been associated with industrial employment in the manufacture of munitions. The precise influence exerted by this industrial work cannot be absolutely asserted. Probably it is the same that such work normally exercises upon males, which is referred to later; but some light is thrown on the case by the data here following:—

| July. | Wages[11]. | Cost of living modified index[11]. | Annual Phthisis mortality per 1,000. |
|---|---|---|---|
| 1914 | 100 | 100 | 1·01 |
| 1915 | 105—110 | 120 | 1·16 |
| 1916 | 115—120 | 135 | 1·18 |
| 1917 | 135—140 | 160 | 1·25 |
| 1918 | 175—180 | 180 | 1·34 |
| 1919 | 210—215 | 185 | 1·02 |
| 1920 | 260 | 220 | 0·89 |

The food position during the war period, especially after control was established, is hardly comparable in regard to wages and cost of living with pre-war times. Nevertheless, so long as the increase of wages lagged behind the increase in cost of living, *i.e.*, until the end of 1918, the mortality from phthisis steadily rose; but in 1919 and 1920, when wages were ahead of cost of living, the phthisis mortality fell with astonishing abruptness, even though in these years the housing conditions of the community were by common agreement accepted to be more inadequate than during the previous thirty years. Here is some evidence for disentangling in the effects produced by poverty that due to the influence of bad housing from that due to feeding; and for justifying the contention that food rather than housing is the important influence in poverty which concerns the prevalence of phthisis.

Table VI.—Comparison of Mortality from Phthisis (Women), 1913-19.*

| Age Group | 1901–10 | 1911–14 | 1913† | 1914 | 1915 | 1916 | 1917 | 1918 | 1919 |
|---|---|---|---|---|---|---|---|---|---|
| 5–10 ... ... | 124 | 125 | 100 | 127 | 125 | 157 | 162 | 173 | 106 |
| 10–15 ... ... | 99 | 106 | 100 | 111 | 118 | 117 | 143 | 151 | 109 |
| 15–20 ... ... | 106 | 108 | 100 | 108 | 124 | 129 | 150 | 161 | 130 |
| 20–25 ... ... | 111 | 108 | 100 | 109 | 110 | 122 | 131 | 152 | 120 |
| 25–35 ... ... | 122 | 106 | 100 | 104 | 109 | 108 | 113 | 131 | 103 |
| 35–45 ... ... | 126 | 107 | 100 | 107 | 114 | 114 | 114 | 120 | 94 |

(i) The rise during the war period was highest, 73 per cent., at ages 5 to 10, the time of life when food privations might be expected to exert most effect; and lowest, 20 per cent., at ages 35 to 45.
(ii) The employment of women on munitions probably affected the mortality adversely from age 15 onwards.
(iii) The fall in 1919 was most pronounced at ages 5 to 10.
(iv) The rapid cessation of employment of older women probably allowed the mortality from ages 25 to 45 to return close to the pre-war figure more rapidly than for ages 15 to 25.

* Annual Report of Chief Medical Officer, Ministry of Health, 1919-20.

† The mortality at each age period is stated for this year at 100; the figures, therefore, are only comparative horizontally.

References.

(6) Brownlee, J. "An Investigation into the Epidemiology of Phthisis in Great Britain and Ireland." Parts I. and II. Report No. 18. Medical Research Committee, 1918.

(7) Pearson, K. "The Fight against Tuberculosis and the Death-rate from Phthisis." Dulau & Co., 1911.

(8) "Decline of Tuberculosis in Germany." Tubercle. March, 1921.

(9) Phillips, C. "Tuberculosis in Poland." The Medical Officer, December 24, 1921.

(10) Collis, E. L., and Greenwood, M. "Health of the Industrial Worker." 1921. J. & A. Churchill.

(11) Bowly, A. L. "Prices and Wages, 1914-20." 1921. Calrendon Press, Oxford.

(*To be concluded.*)

## Figures and Tables

**Diagram 1. f1:**
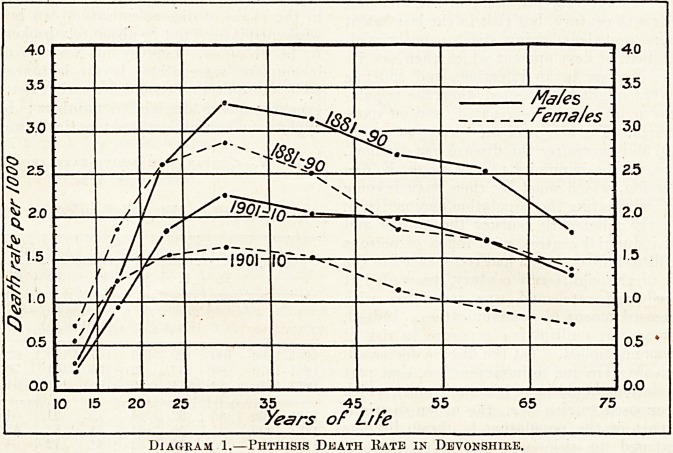


**Diagram 2. f2:**
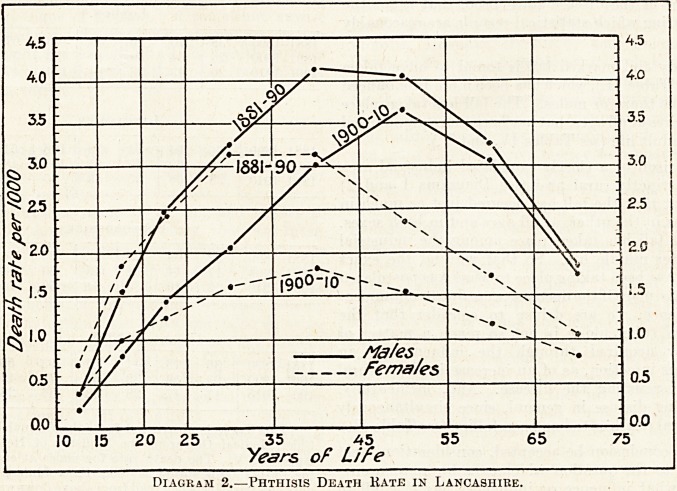


**Diagram 3. f3:**